# Human senescent fibroblasts trigger progressive lung fibrosis in mice

**DOI:** 10.18632/aging.204825

**Published:** 2023-07-01

**Authors:** Fernanda Hernandez-Gonzalez, Neus Prats, Valentina Ramponi, José Alberto López-Domínguez, Kathleen Meyer, Mònica Aguilera, María Isabel Muñoz Martín, Daniel Martínez, Alvar Agusti, Rosa Faner, Jacobo Sellarés, Federico Pietrocola, Manuel Serrano

**Affiliations:** 1 Department of Pulmonology, Respiratory Institute, Hospital Clinic, Barcelona 08036, Spain; 2 Institute for Research in Biomedicine (IRB Barcelona), The Barcelona Institute of Science and Technology (BIST), Barcelona 08028, Spain; 3 Instituto de Investigaciones Biomédicas August Pi i Sunyer (IDIBAPS), Barcelona 08036, Spain; 4 Department of Pathology, Hospital Clinic, Barcelona 08036, Spain; 5 Centro de Investigación Biomédica en Red Enfermedades Respiratorias (CIBERES), Madrid 28029, Spain; 6 School of Medicine, University of Barcelona, Barcelona 08036, Spain; 7 Department of Biosciences and Nutrition, Karolinska Institute, Huddinge 14183, Sweden; 8 Catalan Institution for Research and Advanced Studies (ICREA), Barcelona 08010, Spain; 9 Altos Labs, Cambridge Institute of Science, Cambridge, United Kingdom

**Keywords:** mouse model, cellular senescence, pulmonary fibrosis, antifibrotics, senolytic

## Abstract

Cell senescence has recently emerged as a potentially relevant pathogenic mechanism in fibrosing interstitial lung diseases (f-ILDs), particularly in idiopathic pulmonary fibrosis. We hypothesized that senescent human fibroblasts may suffice to trigger a progressive fibrogenic reaction in the lung. To address this, senescent human lung fibroblasts, or their secretome (SASP), were instilled into the lungs of immunodeficient mice. We found that: (1) human senescent fibroblasts engraft in the lungs of immunodeficient mice and trigger progressive lung fibrosis associated to increasing levels of mouse senescent cells, whereas non-senescent fibroblasts do not trigger fibrosis; (2) the SASP of human senescent fibroblasts is pro-senescence and pro-fibrotic both *in vitro* when added to mouse recipient cells and *in vivo* when delivered into the lungs of mice, whereas the conditioned medium (CM) from non-senescent fibroblasts lacks these activities; and, (3) navitoclax, nintedanib and pirfenidone ameliorate lung fibrosis induced by senescent human fibroblasts in mice, albeit only navitoclax displayed senolytic activity. We conclude that human senescent fibroblasts, through their bioactive secretome, trigger a progressive fibrogenic reaction in the lungs of immunodeficient mice that includes the induction of paracrine senescence in the cells of the host, supporting the concept that senescent cells actively contribute to disease progression in patients with f-ILDs.

## INTRODUCTION

Fibrosing interstitial lung diseases (f-ILDs) constitute a complex and heterogeneous group of diseases characterized by non-resolving pulmonary fibrosis [1]. Idiopathic pulmonary fibrosis (IPF) is the most frequent and representative f-ILD [2, 3]. The pathogenesis of f-ILD is complex and still incompletely understood but cell senescence has recently emerged as a potentially relevant pathogenic player [4–7]. Cell senescence is an adaptation of cells to circumstances of unrepairable cellular damage [8, 9]. The entry in senescence involves a profound rewiring of cellular biology that is largely irreversible, with a permanent exit from the cell cycle (in the case of proliferating cells), the acquisition of stable epigenetic changes, the expansion of the lysosomal compartment and a vigorous Senescence Associated Secretory Phenotype (SASP) [10]. The SASP includes multiple pro-inflammatory and tissue remodelling mediators that can foster a fibrogenic cascade and propagate the senescent phenotype to the surrounding cells [11]. In support of the pathogenic role of the SASP in age-related diseases, senescent cells cause systemic frailty [12] and organ deterioration [13, 14] when transplanted to healthy animals. Moreover, lung fibroblasts isolated from patients with IPF display features of cellular senescence [15]. Senescence-related processes are among the top upregulated transcriptional signatures in IPF alveolar type 2 (AT2) cells [16]. However, the contribution of senescent lung fibroblasts in the initiation and progression of lung fibrosis seen in f-ILD is poorly defined.

We hypothesized that xeno-transplantation of senescent human fibroblasts into the lungs of immunodeficient mice may trigger a fibrogenic reaction. Here we: (1) explored this hypothesis *in vivo*; (2) investigated the potential underlying biological mechanisms *in vitro*; and (3) studied the effects of one experimental senolytic compound (navitoclax) and two anti-fibrotic drugs currently used in the treatment of IPF in humans (nintedanib and pirfenidone), both *in vivo* and *in vitro*.

## RESULTS

### Senescent human lung fibroblasts induce progressive lung fibrosis in mice

**Figure 1:**
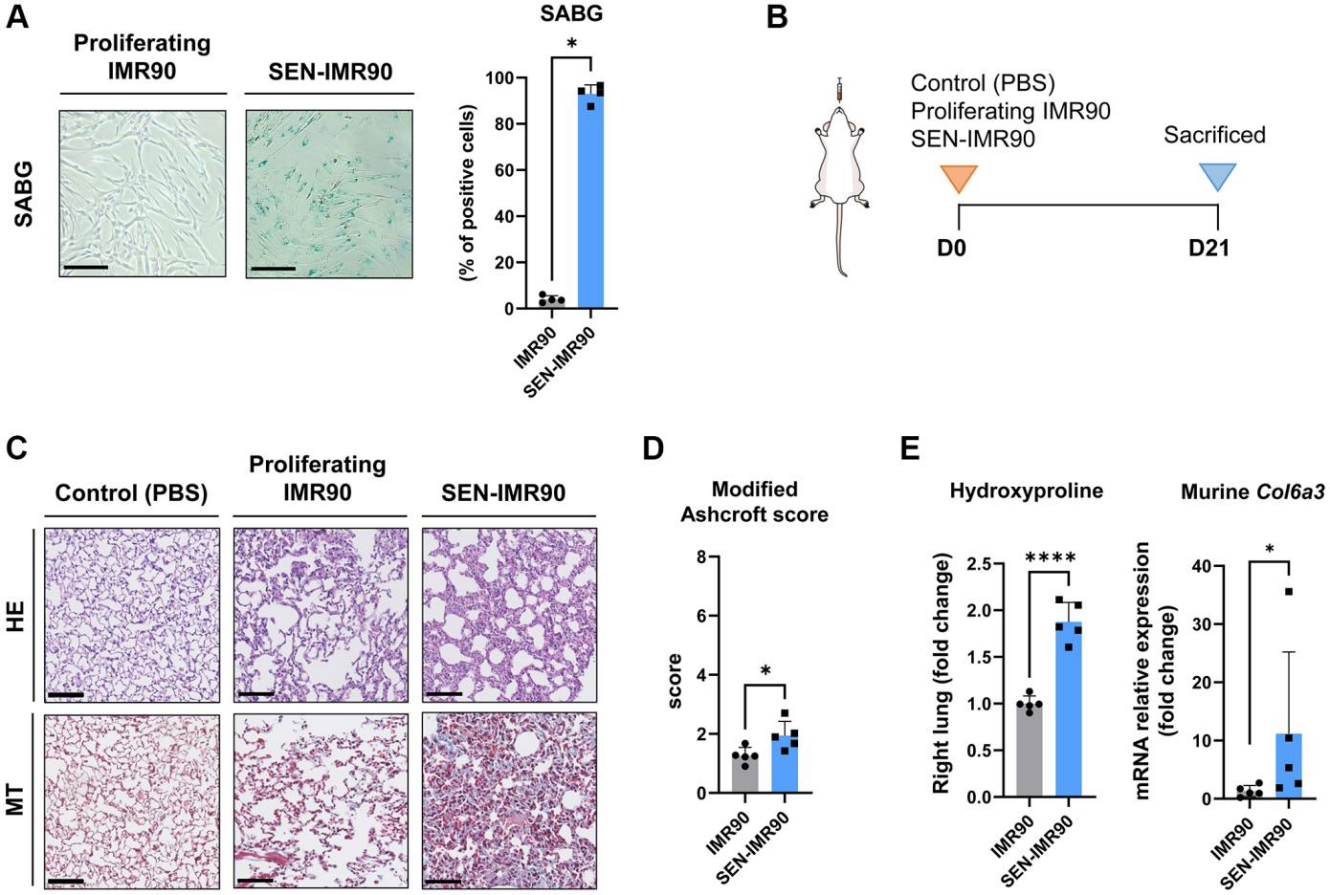
**Senescent human lung fibroblasts induce lung fibrosis in mice.** (**A**) IMR90 lung fibroblasts were exposed to γ-irradiation (20 Gy). Fourteen days later, senescence was confirmed by SABG staining (scale bar, 100 μm). (**B**) Immunodeficient (nude) mice were randomized to receive intratracheal instillation of proliferating human lung fibroblasts (IMR90) or senescent IMR90 (SEN-IMR90). PBS was used as a negative control. (**C**) Representative images of lung sections stained with Hematoxylin Eosin (HE) and Masson’s Trichrome (MT) from mice injected with IMR90 cells, SEN-IMR90 cells or PBS at 21 days post-injection. Scale bar 100 μm). (**D**) Modified Ashcroft score of MT staining in sections from mice injected with IMR90 or SEN-IMR90 cells at 21 days post-injection; *n* = 5. These data are part of a larger experiment presented in Triana-Martinez F, et al. [27]. (**E**) Hydroxyproline content in the right lung tissue of mice injected with SEN-IMR90 compared with IMR90 group at 21 days post-injection; *n* = 5 (left panel). These data are part of a larger experiment presented in Triana-Martinez F, et al. [27]. Relative expression of the mRNA coding for *Col6a3* relative to *Actin-b* levels in lung cell extracts from mice injected with IMR90 and SEN-IMR90 cells at 21 days post-injection; *n* = 5 (right panel). All values are expressed as fold change relative to IMR90 group. Statistical significance was assessed by *U*-Mann Whitney test: ^*^p < 0.05, ^****^p < 0.0001. For further explanations, see text.

As a cellular model, we used human lung fibroblasts (IMR90), which is a normal diploid non-immortalized cell line. Senescence was induced *in vitro* by treatment with γ-radiation as confirmed by detection of senescence-associated beta-galactosidase (SABG) activity, which is a common marker of cellular senescence (Figure 1A). We will refer to these cells as SEN-IMR90. To determine if the xeno-transplantation of senescent human cells in the mouse lungs would initiate a fibrogenic reaction, IMR90, SEN-IMR90, or PBS as control, were intratracheally instilled in the lungs of immunodeficient mice (Figure 1B). After 21 days, collagen deposition, as assessed by Masson’s trichrome (MT) staining, was increased in the lungs from animals xeno-transplanted with SEN-IMR90 cells, but not with IMR90 (or PBS) (Figure 1C). Likewise, the lungs of animals xeno-transplanted with SEN-IMR90 cells had a significantly higher modified Ashcroft score (Figure 1D), higher level of hydroxyproline content, and higher levels of murine *Col6a3* expression (Figure 1E), three well-established markers of fibrosis [17], compared to the IMR90 group. Collectively, these results indicate that human senescent IMR90 cells mediate pro-fibrotic effects in mouse lungs.

### Senescent human lung fibroblasts engraft successfully in mouse lungs

**Figure 2:**
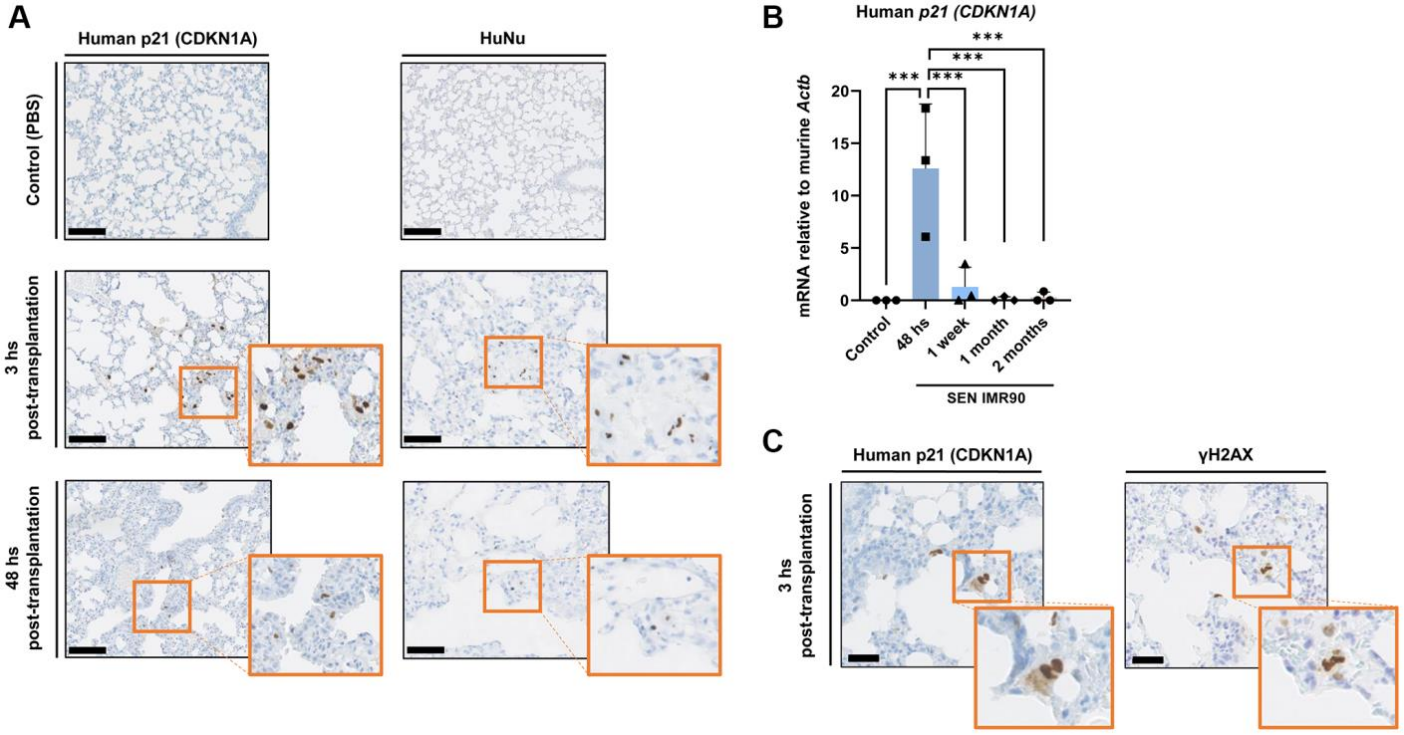
**Senescent human lung fibroblasts infiltration and engraftment in mice lungs.** (**A**) Images of lung sections showing IHC staining for *CDKN1A*/p21^Cip1/Waf1^ (left panel) and HuNu (right panel) from mice injected with SEN-IMR90 or control. Engraftment of senescent cells (arrows) in mice sacrificed after 3 and 48 hours post-transplantation. Positive cells were confirmed using histology at low magnification (20×, scale bar 100 μm), and high magnification (40×, orange box). (**B**) RT–PCR expression of *CDKN1A*/p21^Cip1/Waf1^ was measured relative to murine *Actin-b* to demonstrate the engraftment of SEN-IMR90 in the lungs of nude mice after 48 hours post-transplantation, and their presence at later endpoints compared to control; *n* = 3 each group. Statistical significance was assessed by the one-way ANOVA with Tukey test: ^***^p < 0.001. (**C**) Serial section and staining with antibodies against human *CDKN1A*/p21^Cip1/Waf1^ and Phospho-H2AX (γ-H2AX) to demonstrate senescence state of the engrafted cells. For further explanations, see text.

Based on the pro-fibrotic effects induced by SEN-IMR90 reported above, we explored if these cells can successfully engraft within the lung parenchyma of immunodeficient mice after instillation. We found that human senescent cells, as detected by staining with antibodies against human CDKN1A/p21^Cip1/Waf1^, a senescence marker, and Anti-Human Nuclear Antigen (HuNu), can be localized in the interstitium of mouse lungs 3 hours post-transplantation, and are still detectable 48 hours after instillation (Figure 2A). Similar observations were made when we measured human *CDKN1A*/p21^Cip1/Waf1^ mRNA levels (Figure 2B). We verified senescence in the engrafted cells by serial sectioning and staining with antibodies against human CDKN1A/p21^Cip1/Waf1^ and Phospho-H2AX (γ-H2AX) as a marker for DNA damage and genomic instability (Figure 2C).

### Progression of murine lung fibrosis initiated by senescent human lung fibroblasts

**Figure 3:**
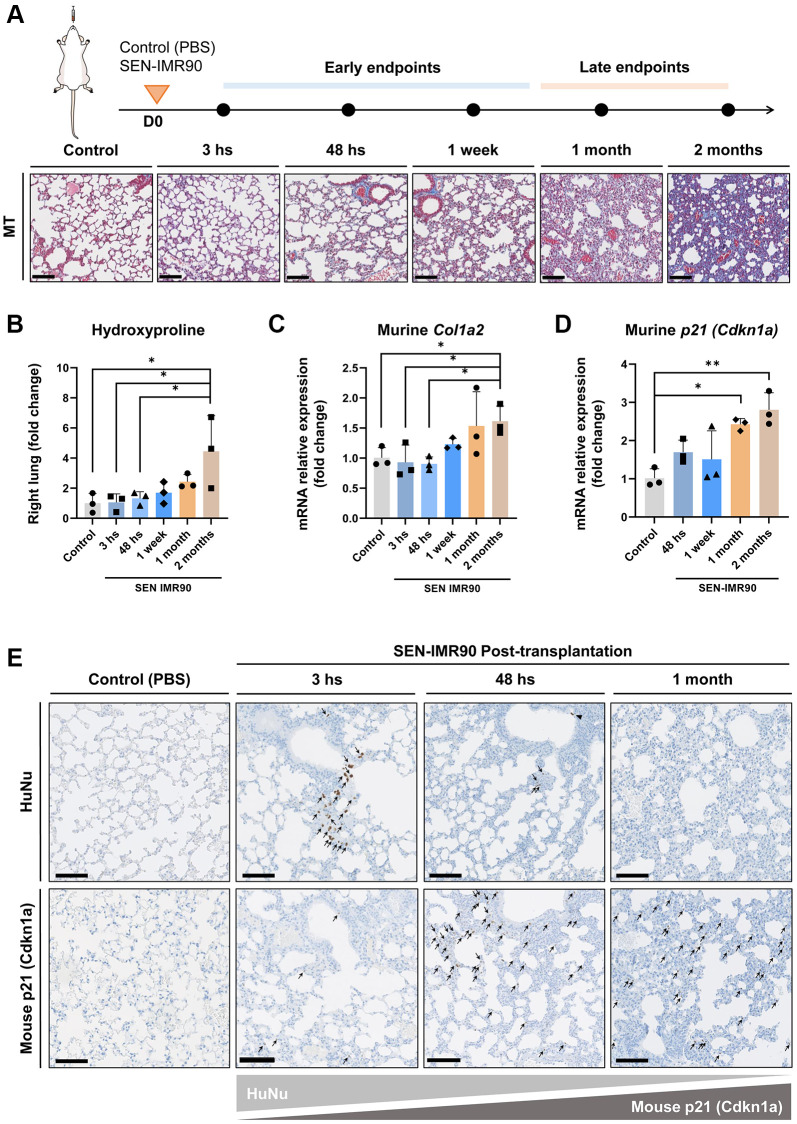
**Lung fibrosis induced by senescent human lung fibroblasts is progressive.** (**A**) Diagram showing the experimental plan to evaluate the dynamics of the development of pulmonary fibrosis in nude mice, as well as representative images of lung sections stained with Masson’s Trichrome (MT) (20×, scale bar 100 μm). These animals received intratracheal instillation of irradiated SEN-IMR90, compared with PBS-exposed mice at early endpoints, and bleomycin-challenged mice (single injection, dose of 3 UI/kg) at late endpoints; *n* = 3 each group. (**B**) Hydroxyproline content in the right lung of mice injected with SEN-IMR90 compared with control (PBS). (**C**) Relative expression of the mRNA coding for murine *Col1a2* in the lungs of the same mice as in panel B. (**D**) Relative expression of the mRNA coding for murine *Cdkn1a*/p21^Cip1/Waf1^ in the same mice as in panel B. For panels B, C and D, *n* = 3 for each experimental group and statistical significance was assessed by the one-way ANOVA with Tukey test: ^**^p < 0.01; ^*^p < 0.05. For panels B, C and D, the group labelled SEN-IMR90 (2 months) is the same group labelled SEN-IMR90 in Supplementary Figure 1A–1C, and the data are the same. (**E**) Images of lung sections showing positive cells using IHC staining for HuNu and mouse p21 (Cdkn1a/p21^Cip1/Waf1^) from mice injected with SEN-IMR90 or control. Engraftment of senescent cells (arrows) in mice sacrificed after different time points (3 hours, 48 hours, and 1 month), showing their dramatic reduction after 48 hours post-transplantation, and the gradually increase of mouse p21 over time (20×, scale bar 100 μm).

To explore how fibrosis evolves over time, we monitored the dynamics of lung fibrosis at different time points after SEN-IMR90 transplant, using PBS as a negative control (Figure 3A). We observed that, over time (up until 2 months after transplant), there was a progressive increase in several fibrotic markers including hydroxyproline content (Figure 3B), and mRNA levels of murine *Col1a2* (Figure 3C) and the murine senescent marker *Cdkn1a*/p21^Cip1/Waf1^ (Figure 3D). This suggests that fibrosis initiated by senescent human cells evolves into murine senescence and fibrosis at late stages of disease. At the latest endpoint analyzed (two months post challenge), fibrosis content was compared to mice treated with a single dose of bleomycin [18, 19]. The levels of hydroxyproline content (Supplementary Figure 1A) and murine *Col1a2* (Supplementary Figure 1B) and *Cdkn1a*/p21^Cip1/Waf1^ (Supplementary Figure 1C) mRNAs were similar in SEN-IMR90 transplanted animals compared to those treated with bleomycin. The increase in murine senescence was confirmed by immunohistochemistry, which showed high levels of p21-positive cells 1 month after instillation of human SEN-IMR90 cells (Figure 3E). This contrasted with the progressive elimination of the engrafted human SEN-IMR90 cells, which were essentially undetectable by qRT-PCR (Figure 2B) or by immunohistochemistry (Figure 3E) 1 month after intratracheal instillation. Therefore, senescent human lung fibroblasts initiate a process of fibrosis that is progressive and associated to an increase in senescent murine cells.

### The secretome of senescent human lung fibroblasts is profibrotic both *in vitro* and *in vivo*

**Figure 4:**
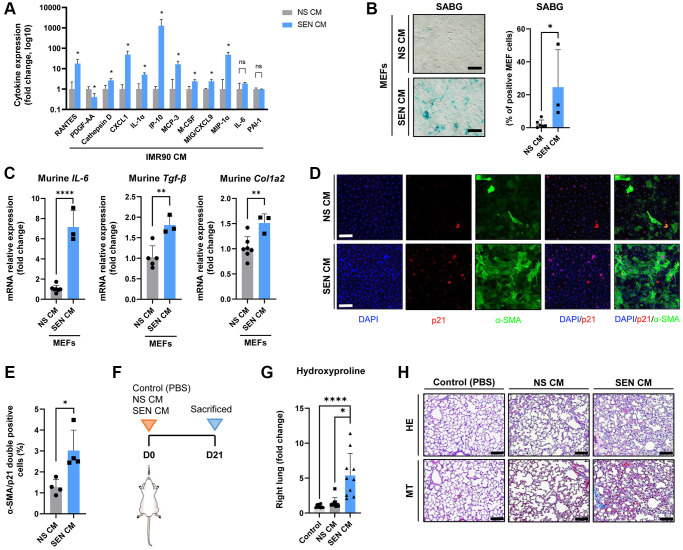
**The secretome of senescent human lung fibroblasts as mediator of murine lung fibrosis.** (**A**) Diagram showing cytokine concentrations in conditioned media (CM) corresponding to 0.5 million cells collected from irradiated SEN-IMR90 (SEN-CM) compared with proliferating IMR90-derived CM (NS-CM) as control, were quantified by using human cytokine arrays; *n* = 4 each group, independent experiments. Statistical significance was assessed by the two-tailed Student’s test: ^*^p < 0.05. (**B**) Mouse Embryo Fibroblasts (MEF) incubated with SEN-CM or NS-CM as control for 6 days. Senescence was confirmed by SABG staining (scale bar, 100 μm). (**C**) Transcriptional upregulation of the profibrotic secretome components (*IL-6, Tgf-β* and *Col1a2*) was confirmed by RT-PCR at 2 days post-exposure to the indicated CM; *n* = 6 SEN-CM and *n* = 3 NS-CM group, independent experiments. Statistical significance was assessed by the two-tailed Student’s test: ^*^p < 0.05. (**D**) Representative immunofluorescence images showing double staining of *Cdkn1a*/p21^Cip1/Waf1^ (red), and α-smooth muscle actin (α-SMA) (green) (10×, scale bar, 100 μm). (**E**) Quantification of the average number of α-SMA/p21 double positive cells as observed in the images shown in panel D using ImageJ. Quantification was performed from 4 experiments with >25 cells quantified for each condition. Statistical significance was assessed by the two-tailed Student’s *t*-test: ^*^p < 0.05. (**F**) Diagram showing the experimental plan to evaluate the effect of the secretome in nude mice lung. These animals were intratracheally delivered SEN-CM or NS-CM, normalized by the number of cells (corresponding to 5 x 10^5^ cells each group), using PBS as negative control; *n* = 10 each group. (**G**) Hydroxyproline content in the right lung tissues of mice injected with SEN-CM or NS-CM, compared with control; *n* = 10 each group. (**H**) Representative images of lung sections of nude mice 21 days after instillation of SEN-CM or NS-CM, or PBS as negative control, stained with Hematoxylin Eosin (HE) and Masson’s Trichrome (MT) (40×, scale bar 100 μm) showing that SEN-CM initiated a cascade of the events that induced mild fibrosis. Statistical significance was assessed by the one-way ANOVA with Tukey test: ^***^p < 0.001; ^*^p < 0.05. For further explanations, see text.

Based on the observations above, we explored the pro-fibrogenic potential of the secretome of human senescent IMR90 lung fibroblasts (SASP), both *in vitro* and *in vivo*. As shown in Figure 4A, we found that the levels of many SASP mediators measured using a multiplex antibody-based commercial assay, including RANTES (Regulated upon Activation, Normal T Cell Expressed and Presumably Secreted), cathepsin D, C-X-C motif chemokine ligand 1 (CXCL1), interleukin (IL)-1α, interferon gamma-induced protein 10 (IP-10), monocyte chemotactic protein 3 (MCP-3), macrophage colony stimulating factor (M-CSF), monokine induced by gamma interferon (MIG), and macrophage inflammatory protein 1-alpha (MIP-1α), were significantly higher (and that of platelet derived growth factor-AA, lower) in conditioned medium (CM) from SEN-IMR90 cells (SEN-CM) compared to CM from non-senescent IMR90 cells (NS-CM), with no significant differences in IL-6 or plasminogen activator inhibitor-1 (PAI-1) levels.

Then, to explore if human SASP factors released by SEN-IMR90 induce senescence in mouse cells, we incubated mouse embryo fibroblasts (MEFs) with CM derived from 6-day cultured SEN-IMR90 and confirmed that it does indeed trigger senescence in MEFs, as indicated by enhanced SABG staining (Figure 4B), and transcriptional upregulation of the profibrotic SASP components *IL-6, Tgf-β* and *Col1a2* (Figure 4C). In addition, we detected by immunofluorescence the expression of CDKN1A/p21^Cip1/Waf1^ and the myofibroblast activation marker alpha smooth muscle actin (α-SMA) (Figure 4D). The amount of double positive α-SMA/p21 cells in MEFs treated with SEN-CM was significantly higher compared to those treated with NS-CM (Figure 4E). Finally, we observed that the instillation of SEN-CM to immunodeficient mice (Figure 4F) triggered a fibrogenic response 21 days after treatment, as indicated by augmented hydroxyproline levels (Figure 4G) and MT staining (Figure 4H).

### Effect of antifibrotic and senolytic drugs

**Figure 5:**
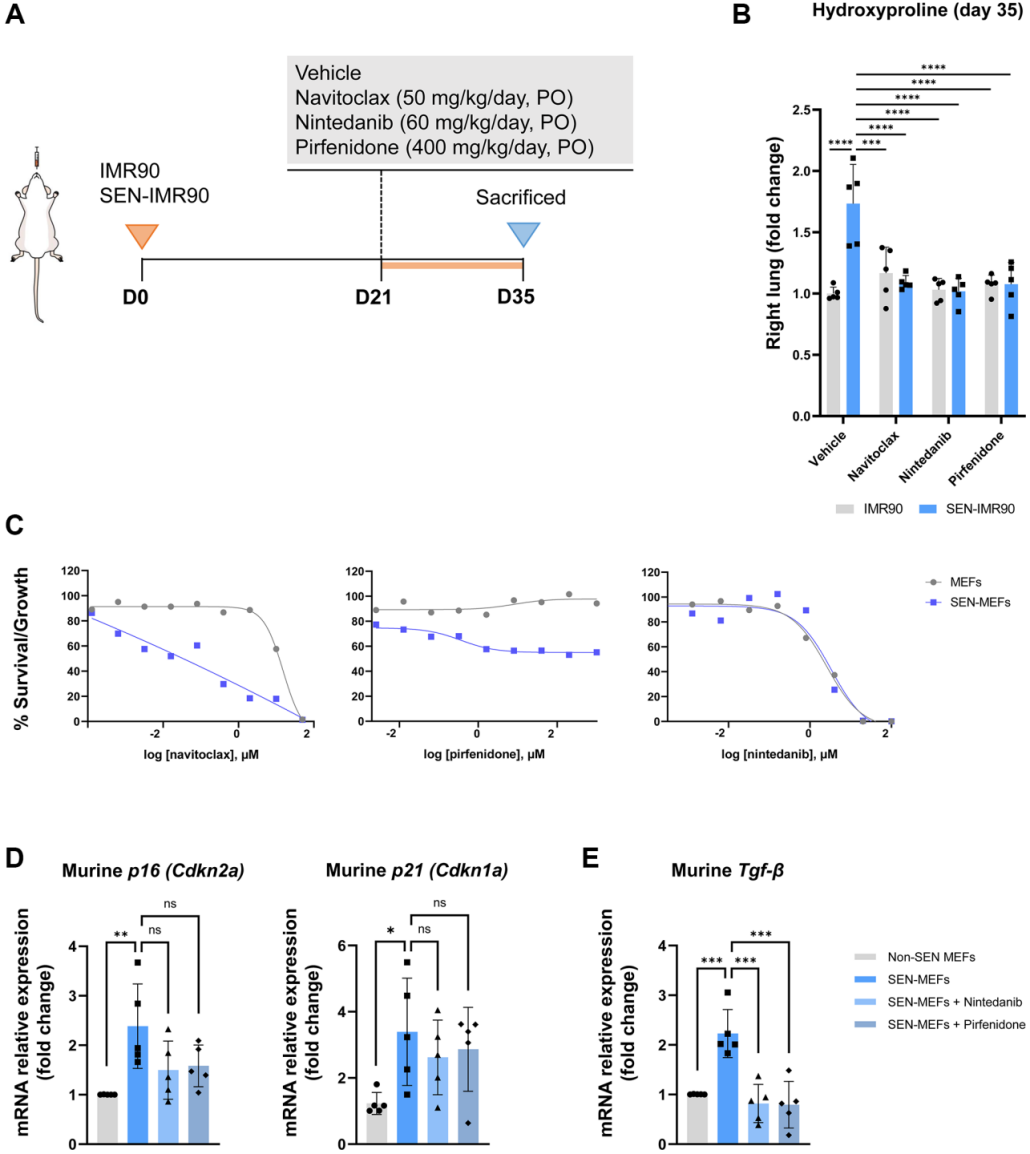
**Effects of antifibrotic and senolytic drugs.** (**A**) Scheme showing the experimental design to assess the effect of antifibrotic or senolytic drugs. Nude mice were randomized after 21 days post-injection of irradiated SEN-IMR90 cells or IMR90 cells as negative control, to either the two approved antifibrotics drugs (nintedanib or pirfenidone), a senolytic drug (navitoclax), or vehicle, for two weeks. (**B**) Hydroxyproline content in the right lung tissues of mice treated with navitoclax, nintedanib or pirfenidone, compared with control; *n* = 5 each group. Statistical significance was assessed by the one-way ANOVA with Tukey test: ^***^p < 0.001; ^****^p < 0.0001. (**C**) Senolytic activity of navitoclax (left panel), pirfenidone (middle panel) or nintedanib (right panel). Diagram showing the senolytic activity of these agents after exposure of senescent MEFs (SEN-MEFs) or non-senescent MEFs (NS-MEFs) to increasing concentration of navitoclax, pirfenidone, nintedanib or vehicle for 72 hours, as confirmed by relative expression of the mRNA coding for murine senescence markers (*Cdkn2a*/p16^INK4a^ and *Cdkn1a*/p21^Cip1/Waf1^), measured relative to *Actin-b* levels in lung cell extracts of nintedanib or pirfenidone group compared to control (**D**); *n* = 5 each group, independent experiments. Statistical significance was assessed by the one-way ANOVA with Tukey test: ^**^p < 0.01; ^*^p < 0.05. (**E**) Relative expression of the mRNA coding for *Tgf-β* (transforming growth factor-β) was measured relative to *Actin-b* levels in SEN-MEFs treated with pirfenidone or nintenadib, compared with control; *n* = 5 each group, independent experiments. Statistical significance was assessed by the one-way ANOVA with Tukey test: ^***^p < 0.001. For further explanations, see text.

Twenty-one days after the instillation of SEN-IMR90 cells (or proliferating IMR90 cells), mice were randomized to receive nintedanib, pirfenidone, navitoclax or vehicle for two weeks (Figure 5A). We observed that hydroxyproline levels (Figure 5B) were significantly lower in mice treated with any of these drugs vs. vehicle.

Since pirfenidone and nintedanib were able to rescue the fibrotic phenotype in mouse lungs initiated by senescent human fibroblasts, we then investigated if these agents had senolytic activity in mouse cells *in vitro*. To this end, we induced senescence in MEFs with bleomycin for 7 days and senescent (and non-senescent) MEFs were then exposed to increasing concentrations of nintedanib, pirfenidone, navitoclax or vehicle for 72 hours. We observed that, in line with its well documented senolytic properties, navitoclax induced apoptosis with higher efficiency in senescent MEFs compared to non-senescent MEFs (Figure 5C). In contrast, nintedanib or pirfenidone were not pro-apoptotic (pirfenidone) or were equally pro-apoptotic over senescent and non-senescent MEFs (nintedanib) (Figure 5C) [20]. In keeping with this observation, neither nintedanib or pirfenidone reduced the levels of the senescent markers *Cdkn2a*/p16^INK4a^ and *Cdkn1a*/p21^Cip1/Waf1^ in senescent MEFs (Figure 5D). Thus, the anti-fibrotic effect of pirfenidone and nintedanib seems unrelated to promoting selective apoptosis of senescent cells in the lung. As anticipated, both drugs decreased the expression of *Tgf-β* in senescent MEFs treated with these molecules (Figure 5E), reinforcing the widely accepted concept that these drugs operate mainly by reducing TGF-β.

## DISCUSSION

The main results of this study are that: (1) human senescent fibroblasts engraft successfully in the lungs of immunodeficient mice and trigger progressive lung fibrosis; (2) the secretome of senescent human lung fibroblasts (SASP) is profibrotic both *in vitro* and *in vivo;* and, finally, (3) navitoclax (an experimental senolytic compound) and two anti-fibrotic drugs currently used in the treatment of IPF in humans (nintedanib and pirfenidone) ameliorate lung fibrosis induced by senescent human fibroblasts *in vivo*, albeit only navitoclax displayed clear direct senolytic activity.

### Previous studies

Existing animal models of f-ILDs do not fully recapitulate the complex pathobiology of human interstitial diseases, thus limiting their use to explore potential candidate therapeutic drugs. For instance, spontaneous resolution of tissue injury is a major drawback of the bleomycin-induced lung fibrosis model, which currently is the most widely employed animal model of IPF [21–26]. On the other hand, cell senescence has been recently identified as a potentially relevant pathogenic mechanism in IPF [15, 16]. Accordingly, we sought to better evaluate the contribution made by senescent human lung fibroblasts toward progressive tissue remodelling observed in fibrotic lung disease. Here we have developed a novel f-ILD experimental model where progressive pulmonary fibrosis in immunodeficient mice is triggered by the intratracheal xeno-transplantation of human senescent fibroblasts. We propose that this new model of fibrosis initiated by human senescent cells in mice may open a window of opportunity for a better understanding of the pathobiological mechanisms underlying f-ILDs in humans, as well as for the preclinical investigation of potential anti-fibrotic candidate drugs [16, 27–31].

### Interpretation of novel findings

The results of this study support the tenet that senescent cells integrating the lung parenchyma are sufficient to recapitulate key pathologic features of f-ILDs and enable the propagation of the senescent phenotype (secondary senescence) at later stages of disease. In particular, we showed that transplanted human senescent cells initiate a process that evolves into murine fibrosis at later stages of disease. This observation reinforces the concept that the senescent secretome causally accounts for the ability of senescent cells to initiate and feed the fibrogenic cascade in the lung [7, 32, 33]. In keeping with this observation, we demonstrate that SASP factors derived from human senescent fibroblasts induce a molecular signature of senescence in mouse recipient cells *in vitro,* while mirroring the pro-fibrotic effect of transplanted human senescent cells in inducing fibrotic damage in the mouse lung. Thus, collectively, our *in vitro* and *in vivo* results pinpoint towards the secretome of senescent human fibroblasts, which is a known source of factors implicated in proliferation and tissue rearrangement in lung fibrosis [7, 32–34], as a mechanism by which these cells mediate a humanized fibrotic pulmonary disorder in mice.

On the other hand, and in keeping with the notion that senolytic drugs may expand the available toolbox to treat pulmonary fibrosis [32], we also showed that the prototypical senolytic drug, navitoclax, reduces the burden of collagen deposition in our experimental model. This is apparently in contrast with the mechanisms of action of two drugs currently used in the clinic to treat IPF (pirfenidone and nintedanib) which also ameliorate lung fibrosis independently of their ability to promote apoptotic death of senescent cells. In agreement with previous reports [35, 36], we show that pirfenidone and nintedanib can reduce the expression of the pro-fibrotic factor TGF-β. Based on these findings, we propose that these two drugs act as “senomorphic” agents by modifying the composition of the SASP [37].

### Potential limitations

We are aware that the method of senescence induction impacts the repertoire of SASP factors expressed, which further adds to the complexity of the SASP which may also be both cell type and microenvironment specific [38]. Our study shows the contribution of the secretory profile of human senescent fibroblasts triggered by γ-radiation, since ionizing radiation-induced senescence is known to cause tissue fibrosis [39, 40]. The diverse biological roles of other senescence inducers (DNA-damaging chemotherapeutic agents, oncogene-induced senescence, or replicative senescence) or cell types in lung fibrosis using this system still need to be investigated, particularly *in vivo*.

Furthermore, we used immunodeficient mice to facilitate the engraftment of human cells in the lung, but we recognize that this may represent a limitation of our model because these mice are unable to mount adaptive immune responses. T cells can be implicated in the pathogenesis of pulmonary fibrosis, can participate in the surveillance of senescent lung cells and may even be involved in the therapeutic pathways of antifibrotic molecules [41]. In this sense, the interaction of the immune response with senescent cells cannot be widely studied with this new model. However, we also showed that our experimental model can be a useful preclinical platform to evaluate antifibrotic therapies, as testified by the fact that two approved drugs for IPF (pirfenidone and nintedanib) significantly improved the fibrotic phenotype initiated by human senescent cells.

## CONCLUSIONS

Our results indicate that human senescent fibroblasts trigger a progressive diffuse mild fibrogenic reaction in the lung of immunodeficient mice through their bioactive secretome. These observations support that accumulation of senescent cells may contribute to fibrotic lung disease in patients with f-ILDs, particularly IPF.

## METHODS

Methods are summarized below and presented in detail in the online supplement.

### *In vivo* humanized mouse model

Human lung fibroblasts (IMR90) were purchased from the American Type Culture Collection (ATCC, Manassas, VA, USA) and grown in Dulbecco’s Modified Eagle Medium (DMEM) (Gibco^®^) supplemented with 10% foetal bovine serum and 100 U/mL penicillin/streptomycin, hereinafter referred to as DM10 media, and maintained at 37°C, 5% CO2. To induce cell senescence, when cells reached 50% confluence, proliferating IMR90 cells were exposed to ionizing γ-radiation (20 Gy). Senescence induction was assessed by monitoring senescence-associated β-galactosidase (SABG) activity using a protocol adapted from Dimri et al. [42]. Then, normal proliferating IMR90 or γ-irradiated senescent human fibroblasts IMR90 (SEN-IMR90) (5 × 10^5^ cells each group) were washed and resuspended in PBS, and then instilled intratracheally to the lung of 6–8 weeks old male nude athymic (*nu/nu*) mice (Envigo Laboratory). At the time points indicated in each experiment, mice were euthanized, and lungs were removed for analysis. The degree of pulmonary fibrosis induced was determined semi-quantitatively by Masson’s trichrome (MT) staining (AR17311-2, Dako – Agilent), and quantitatively by the concentration of lung hydroxyproline, the modified Ashcroft score and the collagen mRNA levels, determined using real-time quantitative PCR (RT-qPCR) (PowerUp^™^ SYBR^®^ Green Master Mix, Applied Biosystems, Foster City, CA, USA) [17, 43].

### Instillation of IMR90-derived conditioned medium into mouse lung

IMR90 and SEN-IMR90 cells were cultured in DM10 medium, at 37°C and under hypoxic conditions (5% CO2). After 14 days, medium was removed and exchanged with 25 ml fresh culture medium, and then collected 24 hrs later. Levels of cytokines, chemokines, and growth factors in conditioned medium (CM) from proliferating IMR90 and SEN-IMR90 were quantitated using Human Cytokine 48-Plex Discovery Assay (Eve Technologies Corporation, Cahada). CM corresponding to 0.5 million IMR90 cells was transferred to mice as detailed in the Supplementary Materials, and PBS was used as negative control (see details in the Supplementary Materials); 3 weeks later lungs were removed and analysed.

### *In vitro* experiments

Mouse embryonic fibroblasts (MEFs) were isolated from 13.5-day C57BL/6J mouse embryos and grown in DM10 media at 37°C and under hypoxic conditions (5% CO2), as detailed in the Supplementary Materials. Senescence was induced by either: (1) paracrine induction by CM from IMR90 control or SEN-IMR90, as detailed in the Supplementary Materials; or (2) addition of 100 μM Bleomycin sulphate (A10152, Sigma-Aldrich) to cell culture for 7 days. The degree of senescence induction was monitored by SABG activity as above [42], gene expression analysis (RT-PCR), or immunofluorescence (against p21) as detailed in the Supplementary Materials.

### Effect of antifibrotics and senolytic treatments

#### In vivo

Twenty-one days after instillation of IMR90 or SEN-IMR90 in 6–8 weeks old male nude athymic (*nu/nu*) mice, treatment with navitoclax (50 mg/kg, oral gavage, 14 days), nintedanib (60 mg/kg, oral gavage, twice per day, 14 days; Boehringer Ingelheim), or pirfenidone (400 mg/kg, oral gavage, twice per day, 14 days; Roche), or vehicle was started, and mice were euthanized thereafter. The degree of pulmonary fibrosis was determined quantitatively by the concentration of lung hydroxyproline.

#### In vitro

To determine the induction of apoptosis after treatment with navitoclax, pirfenidone or nintedanib in control and senescent MEFs, we used CellTiter-Glo^®^ Luminescent Cell Viability Assay (Promega, Madison, WI, USA) or CellTiter-Blue^®^ Cell Viability Reagent (Promega) as detailed in the Supplementary Materials.

### Statistical analysis

Results are presented as mean ± SD. Groups were compared using non-parametric Mann-Whitney *U* test, unpaired Student’s *t* tests or one-way ANOVA with Bonferroni post hoc tests, as appropriate, using Prism 9 software (GraphPad Prism Software, San Diego, CA, USA). A *p* value < 0.05 was considered significant in all cases.

## Supplementary Materials

Supplementary Materials and Methods

Supplementary Figure 1
