# Supplementary material for: Human senescent fibroblasts trigger progressive lung fibrosis in mice
Source: Aging (Albany NY). 2023 Jul 1;15(14):6641–57. doi: 10.18632/aging.204825 (PMC10415539; doi:10.18632/aging.204825)
Supplement: Supplementary Materials and Methods [file aging-15-204825-s001.pdf]

SUPPLEMENTARY MATERIALS AND METHODS

In vivo humanized mouse model

Induction of senescence in cultured human lung fibroblasts

Mouse embryonic fibroblasts (MEFs) were isolated from a 13.5-day C57BL/6J mouse embryo. Cells were grown in Dulbecco's Modified Eagle Medium (DMEM) (Gibco®) supplemented with 10% fetal bovine serum and 100 U/mL penicillin/streptomycin, hereinafter referred to as DM10 media, and maintained at 37°C, 5% CO2. Passaging of cells was performed by enzymatic detachment using 0.05% Trypsin-EDTA (Gibco®) on cells for 5 minutes followed by inactivation in DM10 media and centrifugation at 180xg for 5 min. Supernatant was aspirated to remove dead cells and debris and pellet were resuspended in fresh DM10 media. For *in vivo* experiments, cells were washed and resuspended in PBS before inoculation.

For senescence induction, proliferating IMR90 cells were exposed to ionizing γ-radiation (20 Gy) when they reached 50% confluence. Senescence was induced in MEFs by treatment with 100 μM Bleomycin sulphate (Sigma Aldrich, A10152) for 7 days. For the assessment of optimal senescence induction, senescence-associated β-galactosidase (SABG) activity was monitored using a protocol adapted from Dimri et al. [1]. Briefly, cells were fixed (5 mM EGTA, 2 mM MgCl2, 0.2% (w/v) glutaraldehyde in 0.1M phosphate buffer (pH 7.3)) for 10 min and washed twice with PBS. Staining was performed by incubating cells with an X-gal staining solution (40 mM citric acid, 5 mM potassium hexacyanoferrate (II), 5 mM potassium hexacyanoferrate (III), 150 mM sodium chloride and 2 mM magnesium chloride in 0.1M phosphate buffer (pH 7.3) adjusted to pH 6.0) for 14–18 hrs at 37°C in a non-CO2 regulated incubator. Images were acquired by brightfield microscopy using an inverted microscope (Olympus CKX41) equipped with a digital camera (Olympus DP20).

Instillation of IMR90 fibroblasts into mouse lung

Normal proliferating IMR90 (IMR90) or γ-irradiated senescent human fibroblasts IMR90 (SEN-IMR90) (5×10^5 cells each group) were delivered to the lung of six-to-eight weeks old male nude athymic (*nu/nu*) mice (Envigo Laboratory). At the time points indicated in each experiment, mice were euthanized by cervical dislocation and lungs were removed for further analysis. All mouse procedures were performed in compliance with guidelines established by the Barcelona Science Park's Committee on Animal Care and under the corresponding approved ethics protocol (CEEA-PCB 10884).

Instillation of IMR90-derived conditioned medium into mouse lung

Normal proliferating IMR90 or SEN-IMR90 were cultured in DM10 medium under standard conditions. Twenty-four hours before medium collection, cells were incubated in serum-free DM10 medium. Conditioned medium (CM) was collected and filtered (0.45 μm) prior to buffer exchange procedure (PD MidiTrap™ G-10 column; GE Life Sciences). CM was then lyophilized and stored at −80°C. CM from IMR90 and SEN-IMR90, normalized by the number of cells, was resuspended in sterile PBS on the same day of the experiment. CM derived from IMR90 or SEN-IMR90 corresponding to 5×10^5 cells was delivered intratracheally to six-to-eight weeks old male immunodeficient mice (Envigo Laboratory). PBS was used as negative control. Three weeks after intratracheal instillation, lungs were removed and analyzed.

Senolytic and antifibrotic treatments in vivo

NS-IMR90 or SEN-IMR90 were instilled in the lung of six-to-eight weeks old male nude athymic (*nu/nu*) mice as described above. Twenty-one days after instillation, mice were treated with navitoclax (100 mg/kg, oral gavage, 14 days; ABT-263, Selleckchem, ref.S1001), nintedanib (50 mg/kg, oral gavage, twice per day, 14 days; Ofev®, Boehringer Ingelheim), or pirfenidone (400 mg/kg, oral gavage, twice per day, 14 days; Esbriet®, Roche), or vehicle. At the end of the treatment, mice were sacrificed.

Histologic assessment of mouse pulmonary fibrosis

Left lung tissue was fixed in 10% neutral buffered formalin solution for 24 hours, transferred into tissue cassettes and washed with PBS for at least 24 hours. Tissues were then sent to the Histopathology Facility of the Institute for Research in Biomedicine (IRB, Barcelona) for standard histological procedures. 4–5 μm tissue sections were obtained and stained with Hematoxylin Eosin and Masson's Trichrome (AR17311-2, Dako–Agilent) for the histological analysis. Samples were first examined in blind, and then in an unblinded fashion for confirmation, by a pathologist. Semiquantitative histological scoring of fibrosis in Masson's Trichrome stained sections was determined at 20–40× using the following scale: 1, ×1; 2, ×2; 3, ×3 increase the thickening of alveolar walls; 4, >×3 thickening of alveolar walls and focal areas of single fibrotic masses. In the case of ambiguous scoring, the intervening number was given.

Immunohistochemistry for anti-Human p21$^{WAF1/Cip1}$ (M7202, Agilent), for anti-Mouse p21 clone HUGO

291H/B5 (CNIO) and for anti- Human Nucleoli[HuNu] (Merck, MAB4383) was performed using a Ventana discovery XT at 1:50–1:250, ready to use (RTU) and 1:300, respectively, for 60 min, for anti-phospho H2A Histone Family Member X [gH2AX] (9718, Cellsignaling) using a Leica BOND RX at 1:750 for 120 min and manually for anti-Human Nucleoli [HuNu, NM95] (ab190710, Abcam) at 1:100 and incubated overnight at 4°C. Antibodies were diluted with EnVision FLEX Antibody Diluent (K800621, Dako-Agilent). Antigen retrieval for $p21^{WAF1/Cip1}$ and p21 clone HUGO 291H/B5 was performed with Cell Conditioning 1 (CC1) buffer (6414575001, Roche) and with Ultra Cell Conditioning 2 (CC2) buffer (5279798001, Roche) for HuNu MAB4383. The recombinant Anti-IgG1 + IgG2a + IgG3 antibody [M204-3] (ab133469, Abcam) at 1:500 for 32min was used to enhance specific labelling of $p21^{WAF1/Cip1}$ and HuNu MAB4383. Secondary antibodies used was OmniMap™ anti-Rb HRP (760-4311, Roche) or the OmniMap anti-Rat HRP (760-4457, Roche). Blocking was done with Casein (ref: 760-219, Roche). Antigen–antibody complexes were revealed with ChromoMap DAB Kit (760-159, Roche). Antigen retrieval for gH2AX was performed with BOND Epitope Retrieval 2 – ER2 (AR9640, Leica) buffer for 20 min and with citrate buffer pH6 at 121°C for 20 min for anti-HuNu NM95. Quenching of endogenous peroxidase was performed by 10 min of incubation with Peroxidase-Blocking Solution (S2023, Dako-Agilent). Unspecific unions were blocked using 5% of goat normal serum (16210064, Life technology) with 2.5% BSA (10735078001, Sigma) for 60min. Blocking of unspecific endogenous mouse Ig staining was also performed using Mouse on mouse (M.O.M) Immunodetection Kit – (BMK-2202, Vector Laboratories) incubated 60 min at room temperature. Secondary antibody was a polyclonal Goat Anti-Mouse 1:100 (P0447, Dako, Agilent) incubated for 30 min or the BrightVision poly HRP-Anti-Rabbit IgG, incubated for 45 min (DPVR-110HRP, ImmunoLogic). Antigen–antibody complexes were revealed with 3-3′-diaminobenzidine (K3468, Dako) or with the DAB (Polymer) (RE7230-CE, Leica), with the same time exposure per antibody. Sections were counterstained with Hematoxylin (Dako, S202084) and mounted with Mounting Medium, Toluene-Free (CS705, Dako) using a Dako CoverStainer. Specificity of staining was confirmed by the mouse IgG1, kappa monoclonal [MOPC-21] (ab18443, Abcam), the rabbit IgG (NBP2-24891, Novus biotech) or the rat IgG (6-001-F, R&D Systems) isotype controls.

Image acquisition

Brightfield images were acquired with a NanoZoomer-2.0 HT C9600 digital scanner (Hamamatsu Photonics, France) equipped with a 20× objective. All images were visualized with a gamma correction set at 1.8 in the image control panel of the NDP view 2 U12388-01 software (Hamamatsu, Photonics, France).

Hydroxyproline assay

Right lung lobes were surgically dissected, weighed, placed into 1.5-mL sterile tubes, and snap-frozen. Frozen lung samples were grinded using a liquid-nitrogen filled mortar and pestle. On the day of the assay, thawed and resuspended in 1 mL of distilled water. Tissues were homogenized using a microsample homogenizer (Precellys). 200 μL of 12N hydrogen chloride was added to 200 μL of homogenized tissues. Samples were placed into a preheated oven set to 120°C and incubated overnight. Biochemical quantification of hydroxyproline was performed using a hydroxyproline assay kit (Amsbio®).

RNA extraction and quantitative Real Time-PCR

Total RNA from lung tissue was extracted by mechanical disruption in 1000 μL of TRIzol® reagent (Invitrogen) using a microsample homogenizer (Precellys) according to manufacturer's instructions. RNA concentration was determined using NanoDrop® ND-1000 UV-Vis spectrophotometer at 260nm wavelength. For extraction of total RNA from cells, 6-well plates were scraped in 1000 μL of TRIzol® reagent (Invitrogen). cDNA was synthesized using the SuperScript™ III Reverse Transcriptase (Thermo Fisher, Waltham, MA, USA). mRNA expression analysis was performed using real-time quantitative PCR (RT-qPCR) (PowerUp™ SYBR® Green Master Mix, Applied Biosystems, Foster City, CA, USA) run on a CFX96™ Real-Time PCR Detection system (Bio-Rad). Relative gene expression was determined using ΔΔCt method by measuring RT-qPCR signal relative to signal of housekeeping gene (*Actb*). The RT-qPCR primers used in this study are listed below:

Target	Species	Forward primer	Reverse primer
Actin b	Mouse	ATGGAGGGGAATACAGCCC	TTCTTTGCAGCTCCTTCGTT
Gapdh	Mouse	AACTTTGGCATTGTGGAAGG	ACACATTGGGGGTAGGAACA
Col6a3	Mouse	ACTGGAACCACGGAAGTTCA	GTCACTTCCAACATCGAGGC
Col1a2	Mouse	AGGTCTTCCTGGAGCTGATG	ACCCACAGGGCCTTCTTTAC
Cdkn1a	Mouse	GTGGGTCTGACTCCAGCCC	CCTTCTCGTGAGACGCTTAC
IL-6	Mouse	ACTCACCTCTTCAGAACGA	CCATCTTTGGAAGGTTCAG

Tgf-β	Mouse	CGGAGAGCCCTGGATACCA	ACTTCCAACCCAGGTCCTTC
Pai-1	Mouse	CCAACATCTTGGATGCTGAA	GCCAGGGTTGCACTAAACAT
Cdkn2a	Mouse	TACCCCGATTCAGGTGAT	TTGAGCAGAAGAGCTGCTACGT
α-SMA	Mouse	CCCAAAGCTAACCGGGAGAAG	CCAGAATCCAACACGATGCC
CDKN1A	Human	TGTCCGTCAGAACCCATGC	AAAGTCGAAGTTCCATCGC

In vitro experiments

Cell culture-conditioned medium experiment

IMR90 cells were cultured in DM10 medium, at 37°C and under hypoxic conditions (5% CO2). For senescence induction, cells were irradiated with 10 Gy and cultured 14 more days. Control cells were cultured in parallel with 3-4× sub-passaging steps in between. After 14 days, medium was removed and exchanged to 25 ml fresh culture medium for senescent and for control cells. CM was collected 24 hrs later and used for the medium transfer experiments. MEFs were generated as described previously. Three to four different MEF clones are representing different biological replicates. MEFs were cultured in DM10 medium, at 37°C and under hypoxic conditions (5% CO2). MEFs were seeded reaching 30–40% confluency prior the first medium transfer for paracrine senescence induction. Next day CM from IMR90 control or IMR90 senescent cells was sterile filtered with a 0.2um filter and transferred to the MEFs. Two days later, a second CM transfer was conducted the same way. Cells were grown for another 3 days and then harvested for the according readout, which is gene expression analysis (RT-PCR), SABG staining and immunofluorescence staining.

Immunofluorescence staining in vitro

MEFs grown in a black 96well plate were fixed with 4% PFA in PBS for 10 min at RT and followed by 2 PBS washing steps. Then, the cells were permeabilized by 0.2% Triton incubation for 5 min at RT followed by 2 PBS washing steps. Anti-smooth muscle actin anti-body and (A5228, Sigma) anti-p21 (ab188224, Abcam) were added and cells were transferred to 37°C shaking platform for 30 min. After 2 PBS washing steps MEFs were incubated with the according secondary antibodies (Thermo Fisher, Waltham, MA, USA) and DAPI for another 30 min, shaking at 37°C. Cells were then washed twice with PBS before adding 50% glycerol and storage at 4°C until microscopy was performed. Microscopy was performed within 1 week post staining using an LSM680 ZEISS confocal microscope (10x objective) taking 2-3 independent field acquisitions per 96 well of each condition. Positive cells were quantified using ImageJ software and represented in percentage.

Senolytic assay in vitro

For senescence induction, MEFs cells were treated with 100µM bleomycin sulfate (Sigma Aldrich, A10152) for 7 days. To determine the induction of apoptosis after the treatment with navitoclax, pirfenidone and nintedanib, control and senescent cells were seeded in flat-bottom-clear 96-well plates at a density of 6,000–8,000 and 4,000–6,000 cells per well, respectively, at 37°C in a 5% CO2 humidified atmosphere. The following day cells were treated with serial dilutions of navitoclax, pirfenidone or nintedanib in 0.2% FBS-containing media. Vehicle treatments were used as control for the entire duration of the assay. Viability was assessed 48-72 hs after treatment upon by means of CellTiter-Glo® Luminescent Cell Viability Assay (Promega, Madison, WI, USA) or CellTiter-Blue® Cell Viability Reagent (Promega). Raw data were acquired by measuring luminescence in a VICTOR Multilabel Plate Reader (Pelkin Elmer) or fluorescence at an excitation/emission wavelength of 560 nm/590 nm in an Infinite 200 PRO Multimode Spectrophotometer (TECAN).

SUPPLEMENTARY REFERENCE

1. Dimri GP, Lee X, Basile G, Acosta M, Scott G, Roskelley C, Medrano EE, Linskens M, Rubelj I, and Pereira-Smith O. A biomarker that identifies senescent human cells in culture and in aging skin in vivo. Proc Natl Acad Sci U S A. 1995; 92:9363–67. https://doi.org/10.1073/pnas.92.20.9363 PMID:7568133
