# Supplementary material for: Human senescent fibroblasts trigger progressive lung fibrosis in mice
Source: Aging (Albany NY). 2023 Jul 1;15(14):6641–57. doi: 10.18632/aging.204825 (PMC10415539; doi:10.18632/aging.204825)
Supplement: Supplementary Figure 1 [file aging-15-204825-s002.pdf]

SUPPLEMENTARY FIGURE

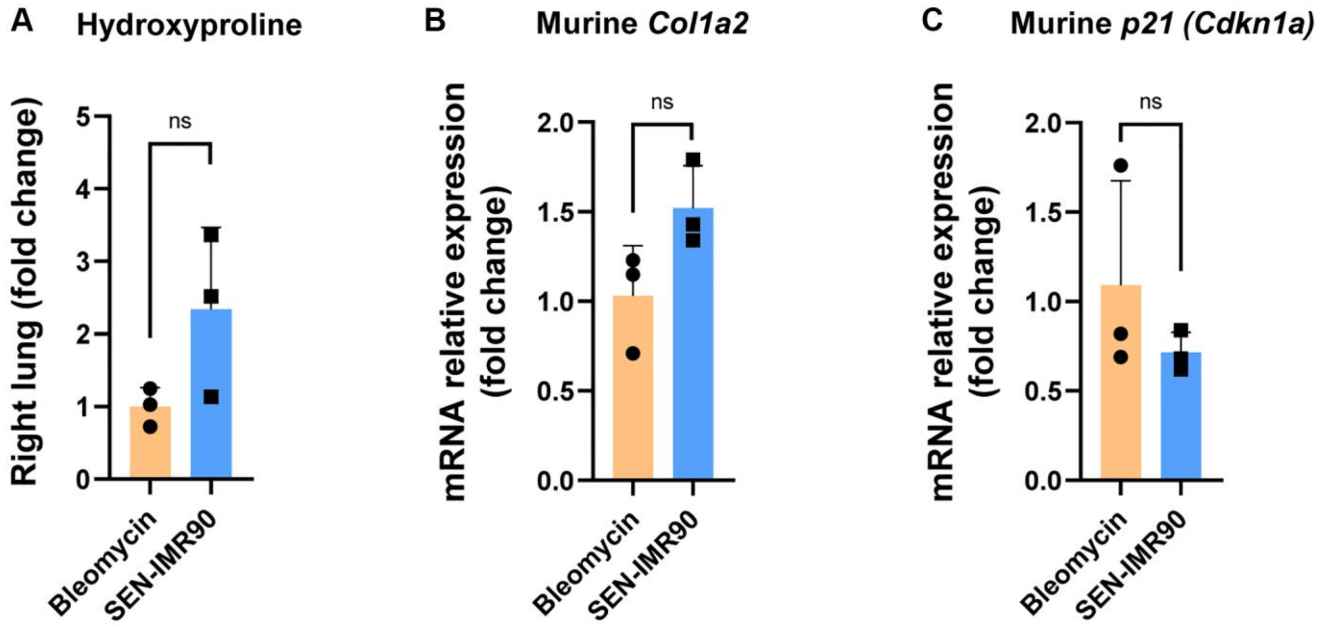

Supplementary Figure 1. Lung fibrosis induced by senescent human lung fibroblasts recapitulates other features of the bleomycin-induced lung fibrosis model. (**A**) Lung fibrosis was ascertained by hydroxyproline assay, and (**B**) mRNA expression of *Col1a2* and (**C**) *Cdkn1a*/p21$^{Cip1/Waf1}$ in lungs of SEN-IMR90 injected mice compared to bleomycin treated mice, 2 months after treatment. For each experimental group, n = 3. The group labelled SEN-IMR90 is the same group labelled SEN-IMR90 (2 months) in Figure 3B, 3C, 3D and the absolute data are the same. Statistical significance was assessed by two-tailed Student´s *t*-test. Abbreviation: NS: not significant. For further explanations, see text.
